# TRAJECTORIES OF PAIN, INSOMNIA, AND DEPRESSIVE SYMPTOMS BEFORE ONSET OF DEMENTIA

**DOI:** 10.1093/geroni/igz038.744

**Published:** 2019-11-08

**Authors:** Minhui Liu, Hanzhang Xu, Junxin Liu, Nancy A Perrin, Sarah L Szanton

**Affiliations:** 1 Johns Hopkins University School of Nursing, Baltimore, Maryland, United States; 2 Duke University School of Medicine, Durham, North Carolina, United States; 3 Johns Hopkins School of Nursing, Baltimore, Maryland, United States

## Abstract

This study examined the trajectories of pain, insomnia, and depression during a four-year period in community-dwelling participants aged 65 or older (N=2,486) from the National Health and Aging Trends Study. Self-reported pain, insomnia, and depressive symptoms were collected at each wave. Dementia status was determined using a modified previously validated algorithm. We analyzed retrospective trajectories of each symptom using mix-effect models and a backward timescale. We also tested for differences in each symptom trajectory between those with and without dementia. Results showed that the trajectory of depression significantly differed by dementia status (Wald χ2=388.34; P<.001). Participants with dementia had persistently higher depression level than those without dementia, particularly during the year before dementia onset. Trajectories of pain and insomnia did not differ before dementia onset. Depression increases risks of dementia and studies with longer length of follow-up are needed to detect significant trajectories of pain and insomnia before dementia onset.

